# Acute Malarial Toxæmia, and the Pathology of Diseases of Malarial Origin

**Published:** 1870-12

**Authors:** 


ACUTE MALARIAL TOXÆMIA, AND THE PATHOLOGY OF DISEASES OF MALARIAL ORIGIN.—In an ably written report on this subject by Dr. H. S. Hudson, of Alabama, the ground is taken that "the rapid and extensive destruction of the red corpuscles and the discharge of their contents into the liquor sanguinis, is the primary pathological lesion" in all malarial diseases. Furthermore, that a comparison of the symptoms and *post mortem* appearances of the body in cases of severe malarial poisoning, with those of the bilious remittent of tropical climates, and malarial yellow fevers, will leave no doubt as to the unity of causation of these diseases; while these diseases are not marked by strict uniformity of symptoms, they are sufficiently so to be called acute malarial toxæmia.

Dr. Hudson gives the history of a case in which the poisoning was so intense as to cause death in about three days, and *post mortem* the tissues were found nearly dry from absence of blood; the viscera were pale and blanched; the bladder contained about ten ounces of very dark, almost black urine, which on examination was found to contain a large amount of hæmatine, casts of tubuli, and a few altered blood globules. The blood was markedly deficient in red corpuscles. This case is typical of its class, and shows the real pathological condition of them all.

Heretofore the primary lesion has been regarded as an effect of organic perturbation, instead of being viewed in its true light.

One reason why this lesion of blood corpuscles has been overlooked, is the fact of the great difference in intensity of these diseases, while in all, the secondary disorders of the visceral organs overshadow the lesion upon which the disorder depends; beside, in a mild case, the retrograde metamorphosis is able to carry off the products of blood disintegration and leave nothing of them discoverable. In the case here recorded, at the autopsy no sufficient lesion of any visceral organ was found to account for the death of the patient. Death occurred when the amount of healthy blood was so far reduced that it was no longer able to supply the system with the oxygen necessary to life. There was evidently a death of the blood in the vascular system as shown by the hæmatine in the serum and urine, and the elimination of it by the kidneys before the debris of the cell membrane appeared in the latter fluid.

The microscope revealed the fact, that what the liquor sanguinis had gained, the red corpuscles had lost; the latter were nearly all colorless and shriveled up. The appearance of the hæmatine in the urine is coincident with distressing pain in the lumbar regions.

Two great dangers to be apprehended are, renal congestion, and obstruction of the ducts of the liver. Protein compounds in excess greatly increase the secretion of bile. By the destruction of the red corpuscles, and the discharge of the globulin into the liquor sanguinis we have this excess.

The increasing proportion of bile in the urine is what, in these diseases, we might expect, as the disorder increases in intensity and the tissues become deluged with bile.

